# Treatment and outcome of IgA nephropathy in children from one single center experience

**DOI:** 10.1186/s12887-023-04195-8

**Published:** 2023-07-26

**Authors:** Youying Mao, Wei Zhou, Zhengyu Zhou, Chenxing Zhang, Jiayao Shen, Lei Yin

**Affiliations:** Department of Nephrology, Shanghai Children’s Medical Center, School of Medicine, Shanghai Jiaotong University, Shanghai, China

**Keywords:** IgA nephropathy, Immunosuppressive treatment, Renal remission

## Abstract

**Background:**

There is no standard recommendation for IgA nephropathy treatment in children.

**Methods:**

This is a retrospective study. From 2012 to 2020, newly diagnosed primary IgAN followed up for at least 1 year were enrolled. The correlation of MESTC scores and clinical index including proteinuria, gross hematuria and renal dysfunction was analyzed. Treatment and clinical response of 6 month, 1year and 3 year at follow up were also analyzed. Complete renal remission was calculated with Kaplan-Meier analysis.

**Results:**

The median follow up was 36 months, from 12 months to 87months in 40 IgAN children. Angiotensin-converting enzyme inhibitor (ACEI) was applied to all patients. 30% received ACEI alone; 15% received glucocorticoids; 37.5% received glucocorticoids plus cyclophosphamide, 17.5% received glucocorticoids plus mycophenolate mofetil. Individuals with diffuse mesangial hypercellularity (M1) were more likely to have nephrotic range proteinuria compared to patients with M0 (80% vs. 20%, P < 0.01). Complete renal remission at 6-month, 1-year and 3-year follow up is 50.25%, 70% and 87.5% respectively. Five-year complete renal remission calculated by Kaplan-Meier analysis is 58.4%. Although without significant difference, there is trend of better survival with complete renal remission in group of nephrotic range proteinuria onset. There is no severe adverse effect.

**Conclusion:**

This study supports the use of glucocorticoids plus immunosuppressive in addition to ACEI in IgA nephrology pediatric patients with proteinuria. We suggest proactive immunosuppressive treatment in IgA nephropathy in children. This is from a single center in China as may not same results in other population.

**Supplementary Information:**

The online version contains supplementary material available at 10.1186/s12887-023-04195-8.

## Background

IgA nephropathy (IgAN) is one of the most common primary glomerular diseases worldwide, particularly in the Asia-Pacific region [1]. The clinical manifestations varies greatly from isolated hematuria to heavy proteinuria, rapid renal loss and chronic renal insufficiency [2]. Up to 30% of all people with IgA nephropathy will eventually develop end-stage kidney disease (ESKD) in Chinese adult population [3]. The prognosis is also varied with a 10-year risk of ESKD varying between 5% and 60% based on proteinuria remission [4]. Persistent proteinuria is independent risk of progressive loss of kidney function [5–7].

There is strong evidence suggesting a benefit of Renin-Angiotensin System (RAS) blockade both in children and adults [8]. For adults if proteinuria stays above 0.75–1 g/d despite at least 90 days of optimized supportive care, glucocorticoid therapy may be considered. Other immunosuppressive therapies are not recommended in IgAN except in the setting of rapidly progressive IgAN [9]. In children, randomized controlled trials (RCTs) and specific expert consensus-driven indications are lacking. Some studies suggest that treatment with glucocorticoids leads to improved kidney survival [10–13].

Here we report a retrospective study of treatment, short-term clinical response and long- term outcome of pediatric IgAN from one single center followed up from 12 month to 87month. It shows favorable prognosis after proactive treatment.

## Materials and methods

### Patients

This is a single-center retrospective study. All patients enrolled in this study were newly diagnosed since 2012 as primary IgAN in Shanghai children’s medical center. IgAN was diagnosed according to renal biopsy. Histology MESTC scores were defined according to Oxford IgAN classification [14, 15]. Secondary IgAN such as Henoch-Schonlein purpura nephritis (HSPN) was excluded. Patients included in this study were followed up at least 1 year.

This study was approved by the Ethics Committee of Shanghai Children’s Medical Center, Shanghai Jiao Tong University School of Medicine, and was in accordance with the principles of the Helsinki Declaration.

### Treatment, definition, data collection and follow-up

Therapy was administered mainly based on proteinuria and renal function. The result of renal biopsy (oxford MEST scores) will be taken into consideration for making treatment decision. MESTC scores were described according to published by IgAN working group as following: mesangial score < 0.5 (M0) or > 0.5 (M1), segmental glomerulosclerosis absent (S0) or present (S1), endocapillary hypercellularity absent (E0) or present (E1), tubular atrophy/interstitial fibrosis < 25% (T0), 26–50% (T1), or > 50% (T2), crescents absent (C0) or crescents < 25% (C1) or crescents > 25% (C2) [14, 15]. Angiotensin-converting enzyme inhibitor (ACEI) was applied to all patients because of proteinuria. In general, patients with moderate proteinuria or heavy proteinuria will be given glucocorticoids (CS), or CS plus immunosuppressive treatment. Mild proteinuria with MESTC scores with any one of M1/S1/C1, will also be given glucocorticoids (CS), or CS plus immunosuppressive treatment. The slightly variation was based on parents’ preference. Therefore there are 4 category therapy administered: (1) ACEI alone; (2) ACEI + glucocorticoids (CS); (3) ACEI + CS + cyclophosphamide (CYP) ± mycophenolate mofetil (MMF) (MMF sequentially applied after CYP); (4) ACEI + CS + MMF. Glucocorticoids was given 1-2 mg/kg (Max. 60 mg prednisone) daily for 1 month, then taped by 5-10 mg ever two weeks, totally for at least 6 month. CYP was given intravenously 500 mg/m^2^ monthly, totally for 6 months. MMF was given 20-30 mg/kg daily, for at least 1 year depending on clinical response.

24-hour protein excretion > 150 mg was defined as proteinuria. Nephrotic range proteinuria, moderate proteinuria and mild proteinuria were defined as daily proteinuria quantification following respectively: >50 mg/kg; 20 mg ~ 50 mg/kg and < 20 mg/kg [16]. Complete renal remission was defined as eGFR > 90ml/min/1.73m^2^, 24-hour proteinuria < 150 mg and RBC < 5/HP in urinalysis.

The following clinical and biological parameters were collected for each patient at time of biopsy: age, sex, weight, height, clinical presentation at disease onset, presence of macroscopic hematuria at diagnosis, systolic and diastolic blood pressure, serum albumin, serum creatinine, estimated glomerular filtration rate (eGFR, Schwartz formula) and 24-hour proteinuria. At follow up of 6-month, 1 year, 2 year, 3 year and last visit, the above parameters were also collected. Pathological parameters of MESTC scores were also included for further analysis.

### Statistical methods

The normally distributed continuous variables were summarized as means ± SDs, whereas non-normally distributed continuous variables were summarized as median (range). Categorical variables were summarized as percentages (%) and were assessed using the Pearson χ2 test or Fisher’s exact test. Graphs were produced using GraphPad Prism version 9.0 for Windows. Renal survival with complete remission was analyzed with a Kaplan-Meier plot and the log-rank test. P values were all two-tailed, and p < 0.05 was considered statistically significant. SPSS version 22.0 statistical software was used for all analysis.

## Results

### Clinical characteristics at diagnosis

From Jan 2012 to Dec 2020, 44 patients were newly diagnosed as IgA nephropathy. Patients followed up at least one year were included. Four patients were lost follow up within one year. Totally 40 patients were enrolled for further analysis. As shown in Table 1, the mean age was 10.6 years, 25 boys and 15 girls. The median follow up was 36 months (12-87months). Six patients (15%) presented with hypertension at diagnosis. Nineteen patients (47.5%) presented with gross hematuria. All of the 40 patients had proteinuria. Thirteen patients (32.5%), 10 (25%) and 17 (42.5%) presented with mild proteinuria, moderate proteinuria and nephrotic range proteinuria respectively. The mean serum albumin was 35 g/L. The mean serum creatinine was 54 umol/L. Five patients presented with decreased eGFR (< 90ml/min/1.73m^2^) at diagnosis.

**Table 1:** Clinical characteristics of patients with IgA nephropathy at time of biopsy

Characteristics	Value
Age (yr), mean ± SD	10.6 ± 3.2
Sex(M:F)	25/15 (62.5%/37.5%)
Follow up (month)	36 (12–87)
Hypertension, n(%)	6 (15%)
Gross hematuria, n(%)	19 (47.5%)
Proteinuria (n/%)(mild/moderate/nephrotic range)	13/10/17 (32.5%/25%/42.5%)
Albumin (g/L)	35 ± 8
Creatinine (umol/L)	54 ± 31
eGFR (n,<90ml/min/1.73m2)	5
Pathological parameters (Oxford-MESTC score) (n/%)	
M0/M1	25/15 (62.5%/37.5%)
E0/E1	16/24 (40%/60%)
S0/S1	15/25 (37.5%/62.5%)
T0/T1/T2	40/0/0 (100%/0/0)
C0/C1/C2	24/14/2 (60%/35%/5%)
Treatment (n/%)	
ACEI alone	12 (30%
ACEI + CS	6 (15%)
ACEI + CS + CYP ± MMF	15 (37.5%)
ACEI + CS + MMF	7 (17.5%)

*M*: mesangial hypercellularity; *E*: endocapillary proliferation; *S*: segmental sclerosis/adhesion; *T*: Tubular atrophy/interstitial fibrosis; *C*: cellular or fibrocellular crescents

*ACEI*: angiotensin-converting enzyme inhibitor; *CS*: glucocorticoids; *CYP*: cyclophosphamide; *MMF*: mycophenolate mofetil

According to Oxford classification scores, M1 was shown in 15 patients (37.5%); E1 in 24 patients (60%), S1 in 25 patients (37.5%), C1/C2 in 14/2 patients (35%/5%); no T1 or T2 detected. For treatment, ACEI was applied to all patients. Twelve patients (30%) received ACEI alone; 6 (15%) received glucocorticoids (CS) therapy; 15 patients (37.5%) received CS plus CYP with or without sequential MMF, 7 patients (17.5%) received CS plus MMF therapy. None of the patients had crescentic GN/ RPGN.

## Histopathologic and clinical index correlation

The correlation of MESTC scores, IgA deposit, C3 deposits in histopathology and clinical index including proteinuria, gross hematuria and renal dysfunction were analyzed. As shown in Table 2, individuals with diffuse mesangial hypercellularity(M1) were more likely to have nephrotic range proteinuria compared to patients with M0 (80% vs. 20%, P < 0.01). Individuals with strong IgA deposits (+++) were all showed nephrotic range proteinuria. Endocapillary proliferation, segmental sclerosis/adhesion, cellular/fibrocellular crescents, or C3 deposits had no correlation with proteinuria. There is no correlation of renal function and gross hematuria between histological index including MESTC and C3/IgA deposits.

**Table 2:** Correlation between histopathologic and clinical variables

	Proteinuria (mild/ moderate/ nephrotic range)	*P* value	eGFR (ml/min/per 1.73 m2 ) > 90/90 − 60/<60	*P* value	Gross Hematuria (no/yes)	*P* value
M (n/%)						
M0	11/9/5 (44%/36%/20%)	< 0.01*	23/1/1 (92%/4%/4%)	ns	12/13 (48%/52%)	ns
M1	2/1/12 (13.3%/6.7%/80%)		12/2/1 (80%/13.3%/6.7%)		9/6 (60%/40%)	
E (n/%)						
E0	6/4/6 (37.5%/25%/37.5%)	ns	14/1/1 (87.5%/6.25%/6.25%)	ns	10/6 (62.5%/37.5%)	
E1	7/6/11 (29.2%/25%/45.8%)		21/2/1 (87.5%/8.3%/4.2%)		11/13 (45.8%/54.2%)	
S (n/%)						
S0	4/4/7 (26.7%/26.7%46.6%)	ns	13/1/1 (86.6%/6.7%/6.7%)	ns	8/7 (53.3%/46.7%)	ns
S1	9/6/10 (36%/24%/40%)		22/2/1 (88%/8%/4%)		13/12 (52%/48%)	
C (n/%)						
C0	9/6/9 (37.5%/25%/37.5%)	ns	22/1/1 (91.6%/4.2%/4.2%)	ns	15/9 (62.5%/37.5%)	ns
C1/C2	4/4/8 (25%/25%/50%)		13/2/1 (81.2%/12.5%/6.3%)		6/10 (37.5%/62.5%)	
IgA deposit (n/%)						
+/++	13/10/13 (36.1%/27.8%/36.1%)	< 0.05*	33/1/2 (91.7%/2.7%/5.6%)	ns	18/18 (50%/50%)	ns
+++	0/0/4 (0%/0%/100%)		2/2/0 (50%/50%/0%)		3/1 (75%/25%)	
C3 deposit (n/%)						
-/+	8/9/10 (29.6%/33.3%/37.0%)	ns	25/1/1 (92.6%/3.7%/3.7%)	ns	14/13 (51.9%/48.1%)	ns
++/+++	5/1/7 (38.5%/7.7%/53.8%)		10/2/1 (76.9%/15.4%/7.7%)		7/6 (53.8%/46.2%)	

*M*: mesangial hypercellularity; *E*: endocapillary proliferation; *S*:, segmental sclerosis/adhesion; *C*: cellular or fibrocellular crescents

* Fisher’s Exact Test

### Treatment and clinical response

As mentioned in methods, ACEI was given to all the patients. In addition to ACEI, other 3 category of therapy was given to patients mainly based on proteinuria level, as well as renal function and MESTC scores will be taken into consideration. In patients with mild proteinuria, 7 (53.8%) received ACEI therapy only. In patients with nephrotic range proteinuria, only 1 patient (5.9%) received isolated ACEI therapy, most received immunosuppressive therapy. As shown in Table 3 the treatment regimen in different groups based on proteinuria, patients with high lever proteinuria were more likely to receive potent immunosuppressive therapy.

**Table 3:** Treatment and response of different groups based on proteinuria level

	Treatment(n/%)					Complete remission (n/total,%)					
Groups	ACEI	ACEI + CS	ACEI + CS + CYP ± MMF	ACEI + CS +MMF	*P value*	CR -6 m	*P value*	CR-1Y	*P value*	CR -3Y	*P value*
Mild proteinuria (n = 13)	7/53.8%	2/15.4%	2/15.4%	2/15.4%	0.055^*^	7/13 (53.8%)	> 0.05	8/13 (61.5%)	0.042^*^	6/7 (85.7%)	> 0.05^*^
Moderate proteinuria (n = 10)	4/40%	2/20%	3/30%	1/10%		7/10 (70%)		10/10 (100%)		5/6 (83.3%)	
Nephrotic range proteinuria (n = 17)	1/5.9%	2/11.8%	10/58.8%	4/23.5%		7/17 (41.2%)		10/17 (58.8%)		10/11 (90.9%)	
Total (n = 40)						21/40 (50.25%)		24/40 (70%)		21/24 (87.5%)	< 0.05^**^

* *CR* complete remission

** Pearson chi-square test

* Fisher’s Exact Test

**Fig. 1:**
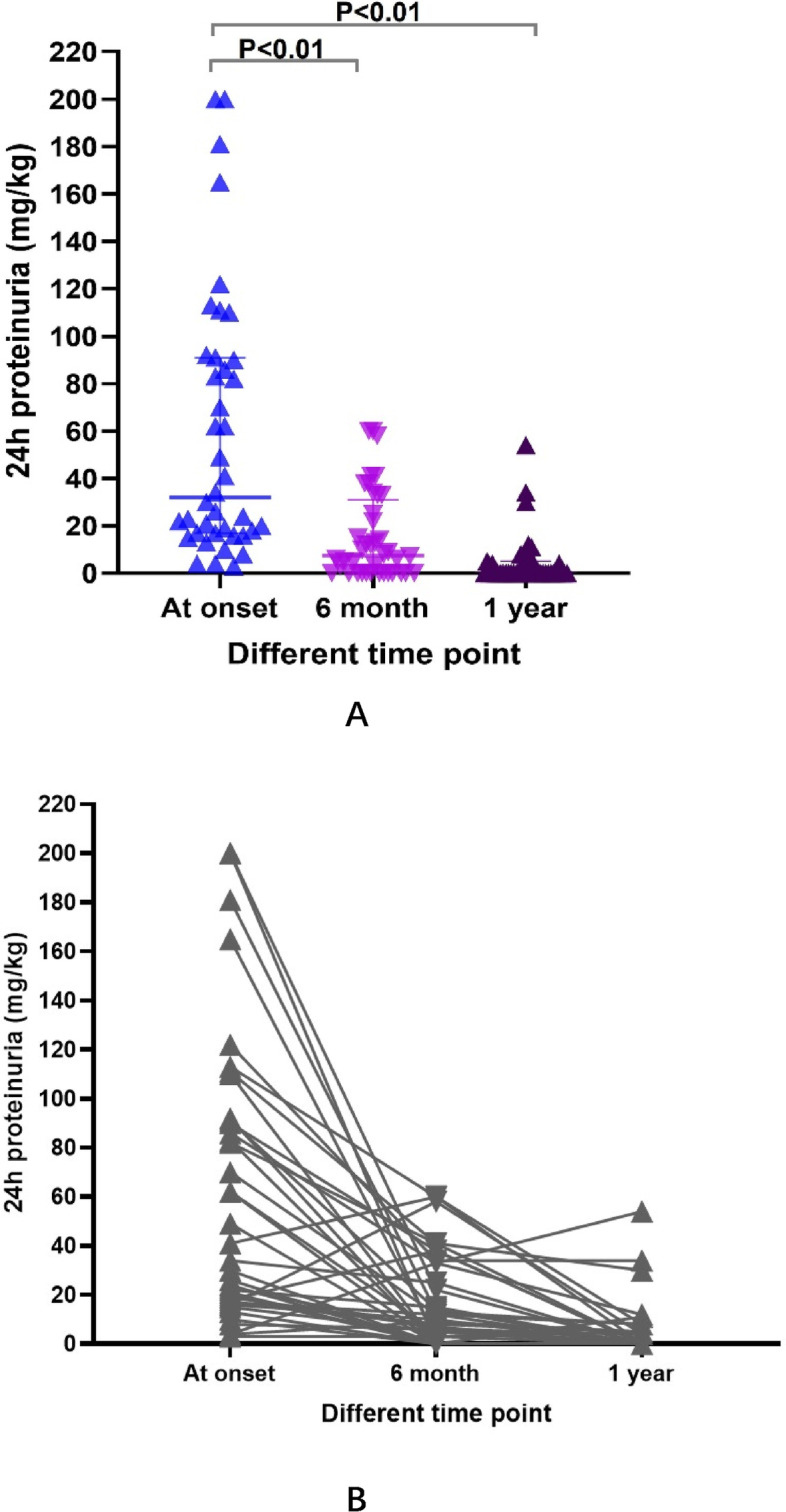
Proteinuria quantification from at onset to 6 month and 1 year after treatment.** A** There is significant decrease of proteinuria quantification at 6 month and 1 year since treatment compared to proteinuria at onset, line presented as median with 25-75% interquartile range. **B** Individual response of proteinuria after 6 month and 1 year treatment, most cases showed decrease after 6 month treatment, while four cases showed opposite way. With prolonger treatment to 1 year, most of the cases (except one case) showed slightly decreased proteinuria or remained stable. Each triangle represents each case

Proteinuria is the main outcome index after treatment. As shown in Fig. 1A, there is significant decrease of proteinuria after treatment at 6 month and 1 year compared to at onset. We also analyzed individual response of proteinuria after 6 month and 1 year treatment, most cases showed decrease after 6 month treatment, while four cases showed opposite way. With prolonger treatment to 1 year, most of the cases (except one case) showed slightly decreased proteinuria or remained stable, Fig. 1B.

At 6-month after treatment, none of patient showed decreased renal function. 53.8% patients with mild proteinuria onset, 70% patients with moderate proteinuria onset and 41.2% patients with nephrotic range proteinuria onset achieved complete renal remission respectively. With prolonged treatment, much more patients achieved complete remission. In group with mild proteinuria at onset, 8 patient (61.5%) achieved complete remission at one year follow up. In group with moderate proteinuria onset, all ten patients (100%) achieved complete remission at one year follow up. 10 patients (58.8%) achieved complete remission in group with nephrotic range proteinuria. There was significant difference of complete remission rate between three different groups at 1 year follow up. Patients with moderate proteinuria at onset had better remission rate at one year treatment. With longer follow up, complete remission of all patients increased to 87.5% at three-year follow up, which was significant higher compared to that at 1 year period, as shown in Table 3. Taken all patients together, complete renal remission at 6 month, 1 year and 3 year follow is 50.25%, 70% and 87.5% respectively.

At 1 year follow up, 12 patients (30%) failed to achieve complete renal remission,9 patients presented with mild proteinuria, 2 patients with moderate proteinuria and 1 patient still with heavy proteinuria. At three-year follow up, 3 patients didn’t achieve complete remission, presented with very mild proteinuria ranged from 223 mg to 300 mg (5 mg/kg – 10 mg/kg), among 3 patients, one accompanied with red blood cell (RBC) 7–8 /HP, and other two were negative in RBC. All the patients showed normal renal function.

International IgA nephropathy pediatric Prediction Tool was used to predict individual disease progression. The median 3-year risk of a 30% decline in eGFR or ESKD of total 40 patients was 13.7% (IQR 10.2–22.4) by international pediatric Prediction Tool. Actually, none of the patients showed decreased renal function at 3 year follow up.

### Complete renal remission calculated with Kaplan-Meier analysis

**Fig. 2:**
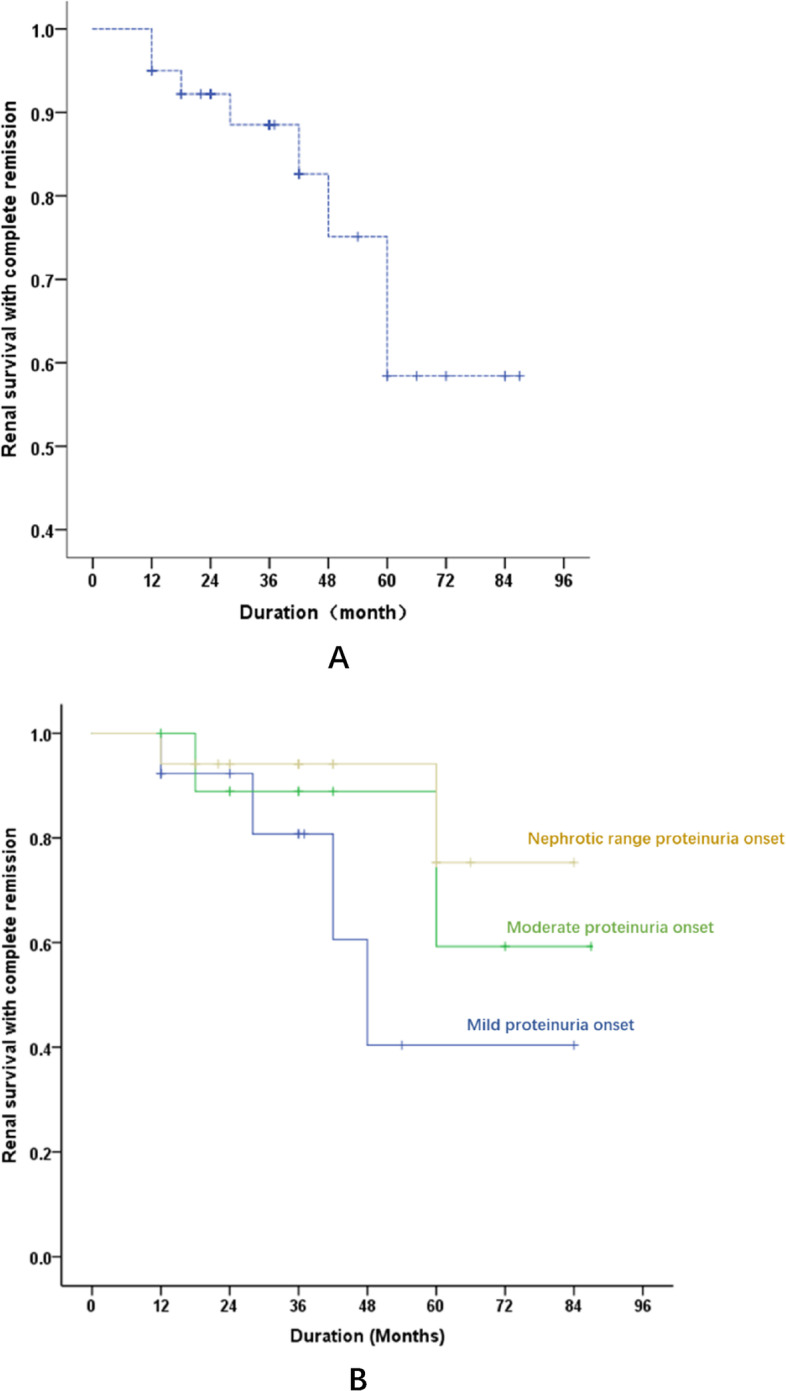
Kaplan-Meier Curve for renal survival with complete remission.** A **Renal survival with complete remission of all patients, 5-year complete renal survival is 58.4%. **B** Renal survival with complete remission compared between different subgroup based on proteinuria quantification at time of renal biopsy. There is trend of better survival with complete remission in group of nephrotic range proteinuria onset, but without significant difference

Complete renal remission was calculated with Kaplan-Meier analysis. As shown in Figs. 2A and 5-year complete renal remission of all patients is 58.4%. This means about 60% patients at 5 year follow up showed no proteinuria, no hematuria or very mild hematuria. Renal survival with complete remission was compared between different subgroup based on proteinuria quantification at time of renal biopsy. As we can see that there is trend of higher complete renal remission rate in group with nephrotic range proteinuria at onset, lower remission rate in group with mild proteinuria at onset, but without significant difference (Fig. 2B).

### Adverse events

Patients treated with glucocorticoids occurred with transient gain of body weight at the first two months. About 15% patients presented with mild intraocular hypertension, they all recovered to normal after lowering intraocular pressure with carteolol eye drops. For patients received CYP, about 10% showed transient decreased CD4 T cell numbers around 3–6 months after the first dose of CYP treatment. There is no severe infection or other adverse events during treatment.

## Discussion

In this study, we reported renal remission and outcome of a pediatric IgAN cohort followed up from 12 to 87months. With potent and advanced treatment, renal remission, short and middle-term renal survival are quite encouraging. None of the patient presented with decreased renal function at the end of follow up. The calculated 5-year complete renal remission is 58.4% by Kaplan-Meier analysis.

IgAN is the most common type of GN worldwide, which has a wide range of prognosis, from microscopic hematuria with stable kidney function through to kidney failure [17–20]. IgAN management should be multifaceted, optimized supportive care, including RAS blockade, blood pressure control, cardiovascular risk minimization, and adherence to lifestyle advice, as suggested by 2021 Kidney Disease Improving Global Outcomes (KDIGO) guideline. If proteinuria stays above 0.75–1 g/d despite at least 90 days of optimized supportive care, the patient has a high risk of progressive loss of kidney function and should be considered for a 6-month course of glucocorticoid therapy [9, 21]. In all patients in whom immunosuppression is being considered, the risks and benefits should be fully discussed. There is evidence from recently RCT studies that adverse treatment effects are more in patients with immunosuppression treatment.

Retrospective study of 1147 patients with IgAN from the European Validation Study of the Oxford Classification of IgAN (VALIGA) cohort showed that immunosuppression was associated with a significant reduction in proteinuria, a slower rate of renal function decline, and greater renal survival [22]. In STOP-IgAN clinical study, addition of immunosuppressive therapy to supportive care was superior to supportive care alone in inducing remission of proteinuria in a proportion of patients, with no change in the rate of decrease in the eGFR. More adverse effects were observed among the patients who received immunosuppressive therapy [23]. With longed follow up, this study failed to detect differences in key clinical outcomes in IgAN patients randomized to receive added immunosuppression on top of supportive care versus supportive care alone [24]. In recent TESTING trial, oral methylprednisolone was associated with potential renal benefit, but with an increased risk of serious adverse events, the trial was early terminated [25]. Taken together, these studies suggest that immunosuppressive therapy may be effective in IgAN, but with appreciable adverse effects [26]. But there was no comparative data for outcomes in IgAN patients based on different proteinuria level and MESTC scores at onset.

The experience of IgAN treatment in children mainly derives from adults. But it is widely acknowledged that treatment of IgAN with immunosuppression differs between adults and children. In children, the use of immunosuppressants is more widespread, particularly the use of glucocorticoids. Yoshikawa reported that in a prospective randomized clinical trial, treatment of children with severe IgA nephropathy with prednisolone, azathioprine, heparin-warfarin, and dipyridamole (combination) for 2 years reduced immunologic renal injury and prevented increase of sclerosed glomeruli, compared to heparin-warfarin, and dipyridamole treatment [13]. With prolonged follow up, combination treatment also improved the 10-year renal survival [27]. Yoshikawa group also compared the effects of prednisolone, azathioprine, warfarin, and dipyridamole (combination) to prednisolone alone in children with IgAN that showed diffuse mesangial proliferation. The percentage of sclerosed glomeruli was unchanged in the patients who received the combination but increased significantly in the prednisolone group [12]. Another retrospective clinical study in IgAN showed similar results that immunosuppressive treatment, especially steroid therapy, seems beneficial in children with glomerular inflammation and proliferation [11]. Different from adults, there are no severe adverse event with steroid and immunosuppressive treatment. All these clinical trials in children demonstrated that steroid and immunosuppressive therapy not only reduce acute injury but also improve long term renal survival without severe side effect.

In this retrospectively study, 70% patients received CS or CS combined with immunosuppressive treatment, Consistent with intense treatment, 70% patients achieved complete renal remission at 1 year since onset of treatment and 87.5% at 3 year. Calculated 5-year complete renal survival is 58.4%. There is trend of better complete renal survival in group of nephrotic range proteinuria at onset, in which group there are more patients received steroid plus immunosuppressive treatment. The result means that patients with nephrotic range proteinuria at onset get more benefits from potent immunosuppressive treatment. None of patients presented with decreased renal function after treatment, which is consistent to previous reported [13]. Renal dysfunction was uncommon after immunosuppressive treatment in IgAN in children. This is the first time to show the outcome of IgAN with intensive immunosuppressive therapy in patients with different proteinuria level at onset.

Severity of proteinuria over time is the most important prognostic indicators of IgA nephropathy [28]. Meta analysis also provided evidence supporting that early reduction in proteinuria at 6 month or 1 year follow up can be used as a surrogate endpoint for studies of CKD progression in IgAN [29]. In this study, at 1 year follow up, 28 patients (70%) achieved complete renal remission, 12 patients (30%) still with proteinuria. Among these 12 patients, 2 patients presented with moderate proteinuria and 1 with heavy proteinuria, 9 patients presented with very mild proteinuria. Based on level of proteinuria at 1 year follow up, we can infer that most of these patients may have very low risk for IgAN progression. The International IgAN Prediction Tool use for children can accurately predict the risk of a 30% decline in eGFR or ESKD in children with IgAN [30]. The median 3-year risk of a 30% decline in eGFR or ESKD in these 40 patients was 13.7% (from 6.2 to 37.1%, IQR 10.2–22.4%) by international pediatric Prediction Tool. Actually, all these 40 patients presented with normal renal function in this study, which maybe benefit from intense CS and immunosuppressive therapy.

There were no severe adverse events. Although renal outcome is very promising in this study, but this is a retrospective study with limited sample size. Longer follow up and more samples are needed to confirm this result.

## Conclusion

In conclusion, this study supports the use of CS plus immunosuppressive in addition to ACEI in IgAN pediatric patients with proteinuria. It is quite safe in children. We suggest proactive immunosuppressive treatment in IgAN in children.

## Supplementary Information

**Additional file 1.**

## Data Availability

The datasets used and/or analysed during the current study available from the corresponding author on reasonable request.
