# Supplementary material for: Treatment and outcome of IgA nephropathy in children from one single center experience
Source: BMC Pediatr. 2023 Jul 26;23:377. doi: 10.1186/s12887-023-04195-8 (PMC10373308; doi:10.1186/s12887-023-04195-8)
Supplement: Supplementary file 1 — Additional file 1. [file 12887_2023_4195_MOESM1_ESM.docx]

**Treatment and outcome of IgA nephropathy in children from one single center experience**

Youying Mao, Wei Zhou, Zhengyu Zhou, Chenxing Zhang, Jiayao Shen, Lei Yin^*^

Department of Nephrology, Shanghai Children’s Medical Center, School of Medicine, Shanghai Jiaotong University, Shanghai, China

^*^Corresponding author: Lei Yin, [scmckidney2022@163.com](mailto:scmckidney2022@163.com);

Abstract

**Background:** There is no standard recommendation for IgA nephropathy treatment in children.

**Methods:** This is a retrospective study. From 2012 to 2020, newly diagnosed primary IgAN followed up for at least 1 year were enrolled. The correlation of MESTC scores and clinical index including proteinuria, mass hematuria and renal dysfunction was analyzed. Treatment and clinical response of 6 month, 1 year and 3 year at follow up were also analyzed. Complete renal remission was calculated with Kaplan-Meier analysis.

**Results:** The median follow up was 36 months, from 12 months to 87months in 40 IgAN children. Angiotensin-converting enzyme inhibitor (ACEI) was applied to all patients. 30% received ACEI alone; 15% received glucocorticoids; 37.5% received glucocorticoids plus cyclophosphamide, 17.5% received glucocorticoids plus mycophenolate mofetil. Individuals with diffuse mesangial hypercellularity (M1) were more likely to have nephrotic range proteinuria compared to patients with M0 (80% vs. 20%, P < 0.01). Complete renal remission at 6-month, 1-year and 3-year follow up is 50.25%, 70% and 87.5% respectively. Five-year complete renal remission calculated by Kaplan-Meier analysis is 58.4%. Although without significant difference, there is trend of better survival with complete renal remission in group of nephrotic range proteinuria onset. There is no severe adverse effect.

**Conclusion:** This study supports the use of glucocorticoids plus immunosuppressive in addition to ACEI in IgA nephrology pediatric patients with proteinuria. We suggest proactive immunosuppressive treatment in IgA nephropathy in children. This is from a single center in China as may not same results in other population.

Key words: IgA nephropathy; Immunosuppressive treatment; Renal remission

**Abbreviation：**

ACEI: Angiotensin-converting enzyme inhibitor

C: cellular or fibrocellular crescents

CS: glucocorticoids

CYP: cyclophosphamide

E: endocapillary proliferation

eGFR: estimated glomerular filtration rate

ESKD: end-stage kidney disease

HSPN: Henoch-Schonlein purpura nephritis

IgAN: IgA nephropathy

KDIGO: Kidney Disease Improving Global Outcomes

M: mesangial hypercellularity

MMF: mycophenolate mofetil

RAS: Renin-Angiotensin System

RCT: randomized controlled trials

S: segmental sclerosis/adhesion

T: Tubular atrophy/interstitial fibrosis

**What is known:**

-No standard recommendation for IgA nephropathy treatment in children.

-Glucocorticoid will be applied in patients with proteinuria after optimized supportive care.

**What is new:**

-A trend of better survival with complete renal remission in IgA nephropathy with nephrotic range proteinuria at disease onset received intensive immunosuppressive therapy.

-The use of glucocorticoids plus immunosuppressive in addition to ACEI showed favorable renal remission.

**Statements and Declarations**

**Ethics approval and consent to participate**: This study was approved by the Ethics Committee of Shanghai Children’s Medical Center, Shanghai Jiao Tong University School of Medicine, and was in accordance with the principles of the Helsinki Declaration. Written informed consent was obtained from the parents.

**Consent for publication**: Not applicable.

**Availability of data and materials**: The datasets used and/or analysed during the current study available from the corresponding author on reasonable request.

**Competing interests**: The authors have no relevant financial or non-financial interests to disclose.

**Funding**: The authors declare that no funds, grants, or other support were received during the preparation of this manuscript.

**Authors' contributions**: Y M, W Z and L Y concepted this study. Y M drafted this article. ZY Z and JY S collected clinical data. CX Z did the statistical analysis. L Y revised this article. All authors read and approved the final manuscript.

**Acknowledgements**: No.
